# Transcriptome analysis of smut fungi reveals widespread intergenic transcription and conserved antisense transcript expression

**DOI:** 10.1186/s12864-017-3720-8

**Published:** 2017-05-02

**Authors:** Michael E. Donaldson, Lauren A. Ostrowski, Kristi M. Goulet, Barry J. Saville

**Affiliations:** 1 Environmental and Life Sciences Graduate Program, Trent University, Peterborough, K9L 0G2 ON Canada; 2 Forensic Science Program, Trent University, Peterborough, K9L 0G2 ON Canada; 3 Present Address: Department of Laboratory Medicine and Pathobiology, Faculty of Medicine, University of Toronto, Toronto, M5S 1A8 ON Canada

**Keywords:** Smut fungi, RNA-seq, Non-coding RNAs, Natural antisense transcripts, *Ustilago maydis*, *Ustilago hordei*, *Sporisorium reilianum*

## Abstract

**Background:**

Biotrophic fungal plant pathogens cause billions of dollars in losses to North American crops annually. The model for functional investigation of these fungi is *Ustilago maydis*. Its 20.5 Mb annotated genome sequence has been an excellent resource for investigating biotrophic plant pathogenesis. Expressed-sequence tag libraries and microarray hybridizations have provided insight regarding the type of transcripts produced by *U. maydis* but these analyses were not comprehensive and there were insufficient data for transcriptome comparison to other smut fungi. To improve transcriptome annotation and enable comparative analyses, comprehensive strand-specific RNA-seq was performed on cell-types of three related smut species: *U. maydis* (common smut of corn), *Ustilago hordei* (covered smut of barley), and *Sporisorium reilianum* (head smut of corn).

**Results:**

In total, >1 billion paired-end sequence reads were obtained from haploid cell, dikaryon and teliospore RNA of *U. maydis*, haploid cell RNA of *U. hordei*, and haploid and dikaryon cell RNA of *S. reilianum*. The sequences were assembled into transfrags using Trinity, and updated gene models were created using PASA and categorized with Cufflinks Cuffcompare. Representative genes that were predicted for the first time with these RNA-seq analyses and genes with novel annotation features were independently assessed by reverse transcriptase PCR. The analyses indicate hundreds more predicted proteins, relative to the previous genome annotation, could be produced by *U. maydis* from altered transcript forms, and that the number of non-coding RNAs produced, including transcribed intergenic sequences and natural antisense transcripts, approximately equals the number of mRNAs. This high representation of non-coding RNAs appears to be a conserved feature of the smut fungi regardless of whether they have RNA interference machinery. Approximately 50% of the identified NATs were conserved among the smut fungi.

**Conclusions:**

Overall, these analyses revealed: 1) smut genomes encode a number of transcriptional units that is twice the number of annotated protein-coding genes, 2) a small number of intergenic transcripts may encode proteins with characteristics of fungal effectors, 3) the vast majority of intergenic and antisense transcripts do not contain ORFs, 4) a large proportion of the identified antisense transcripts were detected at orthologous loci among the smut fungi, and 5) there is an enrichment of functional categories among orthologous loci that suggests antisense RNAs could have a genome-wide, non-RNAi-mediated, influence on gene expression in smut fungi.

**Electronic supplementary material:**

The online version of this article (doi:10.1186/s12864-017-3720-8) contains supplementary material, which is available to authorized users.

## Background

Basidiomycete biotrophic pathogens are a major threat to cereal crop production world-wide. Many of these fungi are obligate biotrophs and not readily amenable to molecular analysis. In contrast, the corn smut fungus *Ustilago maydis* can be cultured in defined conditions, is amenable to biochemical and genetic analysis as well as molecular manipulation, and its genome has been sequenced and annotated [1]. To improve the resources available for using this model, we have created and analyzed deep RNA-seq libraries that will enhance genome annotation. In other classes of phytopathogenic filamentous fungi, similar analyses have enabled the detection of previously unidentified transcripts, and increased the accuracy and coverage of transcriptome annotations. Several studies in the past 2 years alone have employed high-throughput and massively parallel RNA-seq approaches to update current genome annotations of phytopathogenic filamentous fungi including those of *Botrytis cinerea* (504 new gene models; [2]); *Colletotrichum graminicola* (906 new and 819 updated gene models; [3]) and *Fusarium graminearum* (412 new and 1529 updated gene models; [4]). These studies and others have been instrumental in correcting annotation errors, gathering information on untranslated regions (UTRs) and alternative splice sites, as well as identifying novel protein-coding genes and new isoforms, contributing to our understanding of pathogenicity determinants in phytopathogenic fungi. The deep transcriptome analysis presented here involved *U. maydis*, *U. hordei*, and *Sporisorium reilianum* and was carried out with a focus on comparative analyses which expanded the genome annotations and identified conserved natural antisense transcripts.

*U. maydis* was the first biotrophic plant pathogen to have its genome sequenced [1]. This initial sequencing study generated a 20.5 Mb genome sequence containing 6902 protein-coding gene annotations; but, introns were annotated for only 30% of protein-coding genes, and UTR data were not provided. Very few previously-characterized pathogenesis genes were identified, however 426 secreted proteins were predicted. A function could not be ascribed to 298 (70%) of these, and 193 (65%) were found exclusively in *U. maydis*. These secreted proteins included potential effector proteins which stimulated their functional analysis and provided tremendous insight into *U. maydis* pathogenesis (e.g. [5–7]). Further information on secreted proteins was obtained by comparative genome analyses [8, 9], however these comparisons also utilized the initial *U. maydis* genome annotation and did not include information from transcriptome analyses. Earlier expressed-sequence tag (EST) library investigations [10–13] identified novel protein-coding genes, cell-type-specific gene expression profiles, and alternative splicing. They also improved existing gene structure annotations and determined 5’ and 3’ UTR lengths [10, 14]. Together, these transcriptome-focused analyses substantially improved the initial annotation of protein-coding genes in *U. maydis*, providing an improved dataset for functional analysis.

In addition to improving our understanding of the protein-coding genome, EST analyses revealed non-coding transcription in *U. maydis*. Of the 6284 predicted gene locations identified by 24643 ESTs, 800 (13%) aligned to regions of the genome not recognized as coding in the original annotation [10]. Hundreds of non-coding RNAs (ncRNAs), including natural antisense transcripts (NATs), were identified [10, 11, 15]. NATs are long ncRNAs transcribed from the strand opposite to a protein-coding transcript, thus exhibiting sequence complementarity to mRNAs. Several fungal NATs and other ncRNAs are developmentally regulated or expressed in a cell-type-specific manner [15, 16], and functional roles for such transcripts have been elucidated (Reviewed in [17, 18]). Roles in the regulation of pathogenesis and metabolism were revealed by the characterization of a ncRNA and several NATs [11, 15, 19]. The conservation of these transcripts in the related smut species *U. hordei* and/or *S. reilianum*, suggested that conservation of ncRNA/NAT function was also possible [11, 15, 19].

In the past 3 years, transcriptome-wide ncRNA detection in phytopathogenic filamentous fungi has surged with the following findings: 121 ncRNAs in *F. graminearum* [4], 187 small RNAs in *Puccinia striiformis* f.sp. *tritici* [20], 120 ncRNAs in *C. graminicola* [3], 155 ncRNAs in *Rhizoctonia solani* [21], and 5394 ncRNAs in *Zymoseptoria tritici* [22]. Novel NATs have been identified in species including *B. cinerea* (30 spliced NATs; [2]) and *U. maydis* (292 NATs; [15]). In *U. maydis*, altering the level of ncRNAs and NATs through promoter deletion reduced virulence [11, 15]. Together these studies indicate that ncRNAs and NATs contribute to the control of pathogenic development in fungi but provide no information on whether these functions are conserved or unique in different fungal species.

*U. maydis*, *U. hordei*, and *S. reilianum* share common characteristics such as the production of pathogenic dikaryotic filaments as a result of compatible saprophytic haploid cell fusion, proliferation within agronomically important crops, and the production of teliospores as protective dispersal agents [23]. The three species differ in that *U. maydis* infects maize, with localized symptom development [1], whereas *U. hordei* and *S. reilianum* systemically infect barley/oats and maize/sorghum, respectively, eliciting symptoms only in their reproductive structures [8, 9]. Investigations into the molecular basis of these host preference and growth form differences have focused on the identification of distinct effector proteins [12, 16, 17]. However, it is unlikely that this is the only form of variation leading to these differences; here we focus on deep transcriptome analysis of the best characterized smut fungus, *U. maydis*, while augmenting the information obtained through comparative transcriptome analyses. One major difference between these fungi is that *U. maydis* lacks RNAi machinery, while *U. hordei* and *S. reilianum* retain this machinery [9, 24]. Given that Dicer can process long ncRNAs including NATs in filamentous fungi (e.g. [25]), and that long intergenic ncRNAs (lincRNAs) and NATs are known to regulate *U. maydis* gene expression and pathogenesis [11, 15, 19], it is possible that some of the roles of these transcripts in the respective fungi are distinct. If this was the case, these distinct functions could differentially influence gene expression among the smut fungi and contribute to host specificity differences. It is also possible that subtle differences in conserved antisense functions could contribute to variation in pathogenic lifestyle. To begin to investigate these possibilities, the lincRNAs, including NATs in these fungi, must be comprehensively identified.

The existing transcriptome data on *U. maydis*, *U. hordei*, and *S. reilianum* were not collected in a manner that focused on which DNA strands were represented in the transcriptome, nor was there an effort to identify ncRNA expression. The smut fungus transcriptome analyses presented herein utilized massively parallel stranded RNA-seq to update current annotations and provide comprehensive identification and characterization of novel *U. maydis* protein-coding isoforms, lincRNAs, NATs, and their putative orthologs in the other smuts. The identification of a large number of previously unrecognized transcripts provides a critical knowledge base for a more complete understanding of effectors, and the discovery of a large number of lincRNAs and NATs that could influence gene expression and pathogenesis in *U. maydis* as well as the related smut fungi.

## Results

### RNA-seq library creation and RNA-seq based transcript characterization

Compatible haploid cells from each smut species (Table 1) were grown separately, or incubated together to form dikaryons or, for *U. maydis,* co-injected into corn where they fused to form the dikaryon and eventually produced diploid teliospores. RNA was isolated from haploids, dikaryons and teliospores for RNA-seq library creation (Additional file 1). Strand-specific RNA-seq libraries were generated and deep Illumina RNA-sequencing yielded >1 billion paired-end reads (~95 million paired-end reads per library; Additional file 2).

**Table 1:** Fungal strains used in this study

Strain	Genotype	Source
*Ustilago maydis*		
518	*a2 b2*	[59]
521^a^	*a1 b1*	[59]
*Ustilago hordei*		
Uh 4857–4 (alias Uh364)^a^	*MAT-1*	[60]
Uh 4857–5 (alias Uh365)	*MAT-2*	[60]
*Sporisorium reilianum*		
SRZ1	*a1 b1*	[61]
SRZ2^a^	*a2 b2*	[61]

^a^Reference strain used for genome sequencing

Paired-end reads were pooled for each fungus and transfrags were predicted using Trinity [26]. Existing Munich Information Centre for Protein Sequences (MIPS) gene models were updated to include 5’ and 3’ UTRs, alternative splice isoforms, and gene model fusions using the program to assemble spliced alignments (PASA; [27]). Predicted gene models were categorized by Cufflinks Cuffcompare (‘e’ = possible pre-mRNA fragment; ‘i’ = transfrag entirely within reference intron; ‘j’ = potentially novel isoform with at least one splice junction shared with reference transcript; ‘o’ = generic exonic overlap with a reference transcript; ‘p’ = potential polymerase run-on fragment; ‘x’ = antisense transcript; ‘u’ = intergenic transcript). The numbers of identified annotation features for each fungus are presented in Table 2. Detailed genome annotation for each fungus is provided in GFF3-formatted .txt files (Additional files 3, 4 and 5). UTR annotations were determined for 87% of *U. maydis*, 79% of *U. hordei*, and 88% of *S. reilianum* genes; and a low number of UTRs in each of the three fungi contained introns (Table 2). Average UTR lengths (5’/3’) were calculated as 289 bp/304 bp for *U. maydis*, 235 bp/231 bp for *U. hordei*, and 211 bp/180 bp for *S. reilianum* genes. 763, 681, and 466 alternate splice isoforms were predicted using PASA for *U. maydis*, *U. hordei*, and *S. reilianum* genes, respectively (Table 2). The PASA-predicted isoforms differ from those annotated by Cufflinks Cuffcompare ([28]; classes ‘e’, ‘j’, and ‘o’) because by default, PASA was set to ignore alternate splice isoforms whose coding sequence was altered by >30% in length. In an effort to be consistent with previous MIPS gene formatting, these PASA-predicted isoforms were designated as “gene name -N”, where N = an alphabetized letter starting with “-B” (e.g. *UMAG_02775-B*). Further, only 10, 13, and 14 merged transcripts were predicted for *U. maydis*, *U. hordei*, and *S. reilianum* genes, respectively (e.g. denoted as UMAG_*03496: UMAG_03497*). Finally, Cufflinks Cuffcompare was utilized to categorize the remaining PASA assemblies. Novel transcript identifiers were created by combining the code provided by Cufflinks Cuffcompare (described in Table 2) with the corresponding MIPS gene identifier. For example, transcript *x1-UMAG_02150* and *x2-UMAG_02150* represent two unique antisense transcripts to the gene *UMAG_02150*. For the unknown intergenic transcripts (Cuffcompare class ‘u’) >100 nt in length, identifiers were created by combining “u1-” with the PASA assembly identifier (e.g. u1-align_116962). The resulting datasets were used to create updated GFF3-formatted genome annotation files for the three smut fungi (Additional files 3, 4 and 5).

**Table 2:** RNA-seq based predicted gene models

Description	Cuffcompare Code	*U. maydis* ^*a*^	*U. maydis* ^*a*^ with FPKM >1	*U. hordei* ^*a*^	*U. hordei* ^*a*^ *with* FPKM >1	*S. reilianum* ^*a*^	*S. reilianum* ^*a*^ with FPKM >1
MIPS genes and genetic elements		6902		7218		6779	
PASA 5' UTR predictions (contain intron)		6016 (371)		5701 (262)		5971 (239)	
PASA 3' UTR predictions (contain intron)		6064 (146)		5749 (133)		6051 (76)	
PASA predicted transcript isoforms ^b^		763		681		466	
Protein-coding genes and genetic elements		7665	7080 (6394)	7899	6555 (5943)	7245	6585 (6184)
Single exon transfrag overlapping a reference exon and at least 10 bp of a reference intron, indicating a possible pre-mRNA fragment	e	1593 (1423)	330 (298)	1327 (1192)	193 (181)	1003 (910)	183 (172)
A transfrag falling entirely within a reference intron	i	101 (88)	39 (37)	131 (96)	9 (9)	128 (90)	29 (25)
Potentially novel isoform (fragment): at least one splice junction is shared with a reference transcript	j	659 (327)	386 (242)	407 (280)	249 (191)	337 (246)	196 (157)
Generic exonic overlap with a reference transcript	o	227 (202)	115 (107)	164 (152)	81 (78)	133 (130)	64 (62)
Possible polymerase run-on fragment (within 2Kbases of a reference transcript)	p	1875 (1287)	755 (650)	1413 (1055)	473 (423)	1435 (1026)	554 (490)
Exonic overlap with reference on the opposite strand	x	8173 (3469)	4500 (2624)	5847 (2640)	2332 (1606)	6842 (3083)	2768 (1949)
Unknown, intergenic transcript	u	3853 (3853)	1832 (1832)	2313 (2313)	898 (898)	2888 (2888)	1331 (1331)
Total Transcripts		24146	15,037 (12184)	19501	10790 (9329)	20011	11710 (10370)

^a^Multiple transcripts annotated by PASA and Cufflinks Cuffcompare can hit the same gene or genetic element; therefore, non-redundant gene hits are indicated in parentheses

^b^Using default settings, PASA only predicted novel transcript isoforms that did not alter the coding length by >30%

For each RNA-seq library, paired-end reads were mapped to their corresponding genome and updated GFF3 annotation file using CLC Genomics Workbench. Fragments per kilobase of transcript per million mapped reads (FPKM) values were calculated by CLC Genomics Workbench for each transcript. Maximum FPKM values were determined for each transcript across the RNA-seq libraries for *U. maydis*, *U. hordei*, and *S. reilianum*. These maximum FPKM values were then used to estimate the number of detected transcripts using a range of FPKM cut-off values (Additional file 6). Similar to other studies [29–36], a conservative threshold of FPKM >1 was used as the cut-off to indicate whether a transcript was detected by RNA-seq and considered to be expressed by *U. maydis*, *U. hordei*, and *S. reilianum* cells from which RNA was isolated. If the FPKM value for a given transcript was below 1, the transcript was considered to be predicted but not detected. Using these designations, RNA-seq-predicted gene models and RNA-seq-detected gene models were determined for *U. maydis*, *U. hordei*, and *S. reilianum*, respectively (Additional files 7, 8 and 9, summarized in Table 2). For each smut, alternatively spliced isoforms were detected in addition to those predicted by PASA. Overall, this study dramatically increases the number of known transcriptional units for each fungus, as highlighted by the observation that the total number of transcripts detected by RNA-seq (FPKM >1) in this study were 2.2 times the number of *U. maydis*, 1.5 times the number of *U. hordei*, and 1.7 times the number of *S. reilianum* previously predicted gene models.

### Detection of non-coding intergenic transcripts and previously uncharacterized ORFs

A number of intergenic transcripts (class ‘u’ and class ‘p’) were predicted and detected for each smut. Using these nucleotide sequences, the Trinotate annotation suite predicted full-length (“complete”) protein-coding ORFs, and translated those ORFs into peptides for the purposes of identifying similar peptides in known databases using BLASTp, and for locating putative secretion signals using SignalP (Additional files 10, 11 and 12; summarized in Table 3). A small number of intergenic transcripts are predicted to encode proteins and some of these contain secretion signals; however, it is striking that 2414 *U. maydis*, 1206 *U. hordei*, and 1776 *S. reilianum* transcripts represent novel lincRNAs. Further, RNAmmer analysis of the detected intergenic transcripts identified only one new *S. reilianum* rRNA prediction (u1-align_119627). BLASTn comparisons identified lincRNAs with similar sequences among the different smut fungi, indicating some lincRNAs are conserved among the smuts examined in this study (Additional file 13).

**Table 3:** Trinotate results summary for intergenic transcripts

		Peptides >30 aa <100 aa			Peptides >100 aa		
Fungus	Transcripts detected	Peptides^a^	BLASTp hits	SignalP predictions	Peptides^a^	BLASTp hits	SignalP predictions
*U. maydis* (class 'u')	1832	113	13	6	28	12	0
*U. maydis* (class 'p')	755	21	5	0	11	7	0
*U. hordei* (class 'u')	898	58	7	0	26	11	3
*U. hordei* (class 'p')	423	29	2	0	2	0	0
*S. reilianum* (class 'u')	1331	28	0	0	3	1	0
*S. reilianum* (class 'p')	490	10	1	1	4	2	1

^a^Only peptides with a "complete" open reading frame (methionine-stop codon) and a transcript FPKM > =1 are reported

### Antisense transcription is prevalent and conserved among smut fungi

**Fig. 1:**
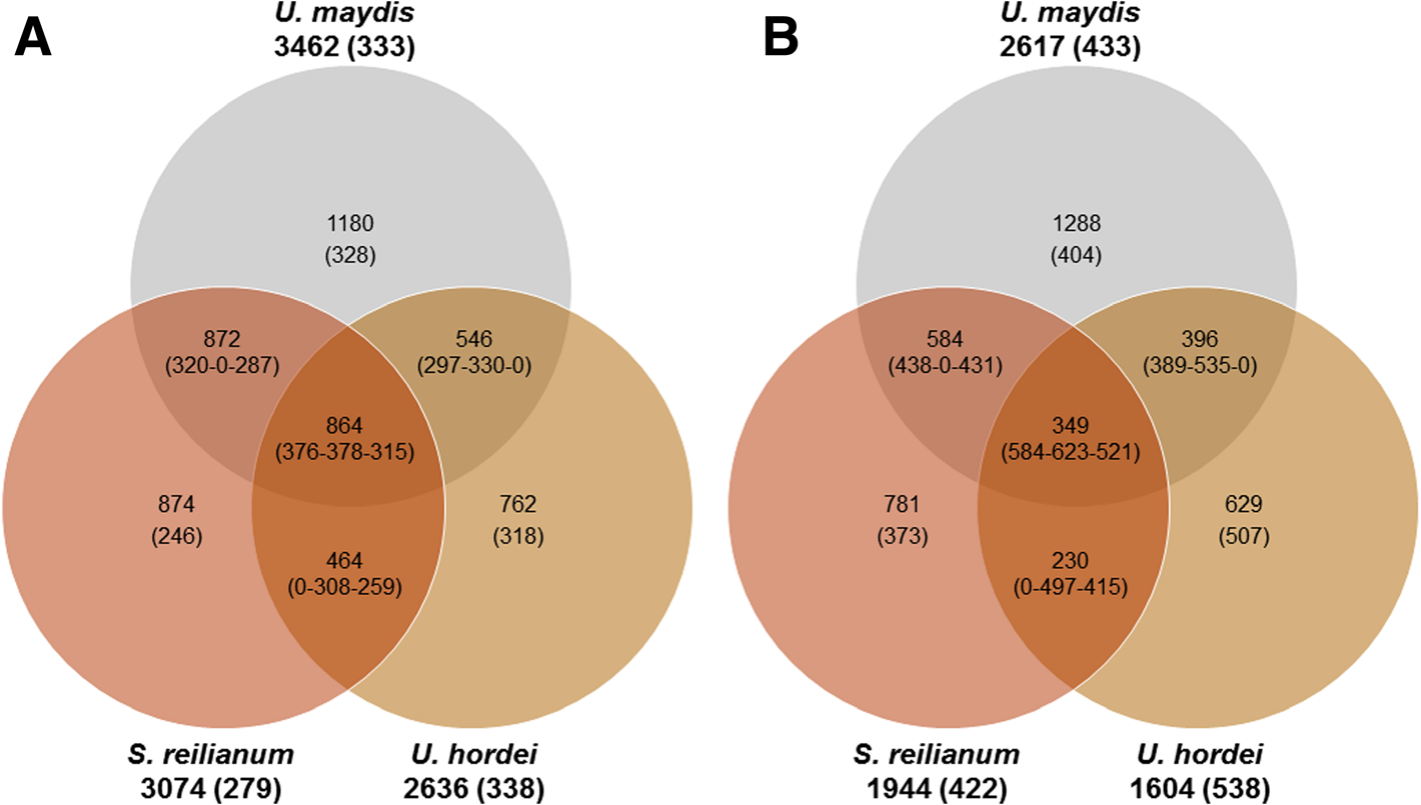
Antisense transcript targets conserved in smut fungi. The number of non-redundant genes with antisense are reported with average antisense lengths (Um-Uh-Sr) for (**a**) predicted or (**b**) detected antisense transcripts. Orthologous loci identities were based on reciprocal best SIMAP hits

Natural antisense transcripts (class ‘x’) were detected for 2624 *U. maydis*, 1606 *U. hordei*, and 1949 *S. reilianum* genes. The predicted NATs are presented in Additional files 14, 15 and 16 and the detected NATs in Additional files 17, 18 and 19. Interestingly, antisense transcripts were consistently longer in *U. hordei* (average length of 538 bp), compared to *U. maydis* (433 bp), and *S. reilianum* (422 bp; Fig. 1). Further, 186, 101, and 34 antisense transcripts were detected that contain introns for *U. maydis*, *U. hordei*, and *S. reilianum*, respectively (Additional files 7, 8 and 9). There was also cell-type-specific expression of NATs in *U. maydis*. NATs complementary to 1284 genes were detected in the dormant teliospore and 657 of these were not detected in haploids or the dikaryon. Similarly, NATs complementary to 164 genes were detected only in the haploid cells and 164 only in the dikaryon. Comparison analysis identified antisense transcripts that were conserved in two or all three of the smut fungi (Fig. 1). Gene Ontology (GO) term enrichment analyses on the 2617 genes with NATs in *U. maydis* revealed enrichment of the molecular function category binding (*p* = 0.0405) and GO analysis of genes complementary to the 349 NATs conserved among all three smut fungi revealed enrichment in the molecular function categories: binding (*p* = 0.00128), DNA binding (*p* = 0.0188), nucleoside triphosphatase activity (*p* = 0.0382), and its sub-category helicase activity (*p* = 0.0154).

### Validation of RNA-seq data

**Fig. 2:**
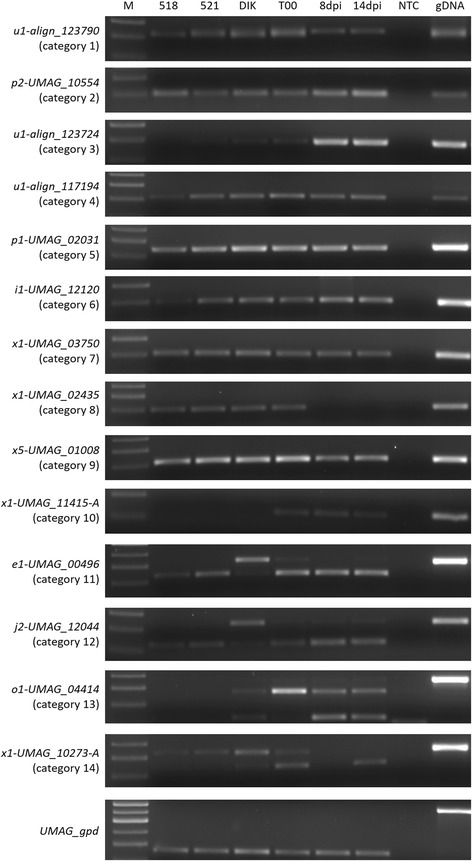
RT-PCR supports RNA-seq-predicted transcripts. RNA was isolated from haploid cells (518, 521), dikaryotic mycelia (UmDIK), dormant teliospores (T00), and *U. maydis*-infected seedlings at 8, or 14dpi and used as template for RT-PCR. Oligo-(dT)_16_, DEPC-treated water or a strand-specific primer was used to prime the reverse transcription reaction. Only the transcript-specific reaction is shown above, along with a genomic DNA PCR control (gDNA) and a no template control (NTC). A size marker was also included (M)

A number of *U. maydis* transcripts detected by RNA-seq were chosen as candidates for expression pattern confirmation tests. Transcripts with similar annotation features, such as cell-type-specific expression or the presence of an intron, were categorized and independently assessed by RT-PCR in 6 distinct *U. maydis* cell-types. Of the 62 candidate transcripts tested, 61 were detected in at least one cell-type. An intron was detected in at least one cell-type of each candidate antisense transcript tested with predicted introns (*n* = 5). Annotation of transcripts from class ‘e’, ‘j’, and ‘o’ suggested the presence of two isoforms. Of these transcripts tested (*n* = 8), two isoforms were detected, with the exception of the *j1-UMAG_11139* and *j2-UMAG_03704* loci where only a single major isoform was found. Examples of transcript confirmations from each category are shown in Fig. 2. Candidate transcripts and overall RT-PCR expression results are summarized in Additional file 20.

## Discussion

In this study, we present deep RNA-seq and genome annotation updates for three closely related smut fungi: *U. maydis*, which causes common smut of corn, *U. hordei*, the causal agent of covered smut of barley, and *S. reilianum*, which causes head smut of corn. These phytopathogens are threats to cereal crop production worldwide and *U. maydis* has become the model for biotrophic plant pathogenesis. Previous genome annotations for these fungi were based on less comprehensive technologies (e.g. EST analysis) and lacked extensive data on important annotation features such as UTR length, alternative splicing events and non-coding transcription (intergenic and antisense). Our massively parallel stranded RNA-sequencing experiments update and expand *U. maydis*, *U. hordei*, and *S. reilianum* genome annotations by including data for these features and uncovering conserved NATs and lincRNAs that can influence gene expression on a genome wide level.

### Updating protein-coding annotations

The genome annotations currently available in the MIPS database represent 6902, 7218, and 6779 protein-coding genes in *U. maydis*, *U. hordei*, and *S. reilianum*, respectively. Our analyses permitted us to update thousands of these gene models (Additional files 7, 8 and 9), which included the identification of: transcript isoforms (predicted by PASA to vary by <30% from existing gene models), pre-mRNA fragments (Cuffcompare class ‘e’), transcripts spanning introns (Cuffcompare class ‘i’), alternatively spliced isoforms (Cuffcompare class ‘j’), transcripts overlapping exons (Cuffcompare class ‘o’), and putative polymerase run-on events (Cuffcompare class ‘p’). Table 2 contains a summary of the number of predicted gene models. To confirm our *in silico* predictions, we screened 62 candidate transcripts in these categories by semi-quantitative RT-PCR. We were able to detect 5/5 pre-mRNA fragments, 3/3 intronic transcripts, 3/5 novel splice isoforms, 5/5 exon overlapping transcripts, and 11/12 putative polymerase run-on transcripts (Additional file 20). The “run-on” transcripts were detected by amplification of an RNA fragment that could also represent separate transcripts. Given that the average intergenic lengths are 1127 bp for *U. maydis*, 1186 for *U. hordei*, and 929 bp for *S. reilianum* [9], the putative polymerase run-on transcripts that are <2 kbp from a neighbouring gene may actually represent separate transcripts. In addition, we updated UTR annotations for 87% of *U. maydis*, 79% of *U. hordei*, and 88% of *S. reilianum* MIPS gene models, for which 5’ UTRs average 289 bp, 235 bp, and 211 bp, and 3’ UTRs average 304 bp, 231 bp, and 180 bp, respectively.

The identification of alternative splicing events in all three smuts is noteworthy. Previous research identified significant rates of alternative splicing across the genomes of several ascomycetes and basidiomycetes including *U. maydis*, and suggested that there may be links between alternative splicing and pathogenesis [37]. The rates of alternative splicing they reported were higher in pathogenic fungi (7.6%) than non-pathogenic fungi (5.1%), although not significantly so. Also, the Pfam domain descriptions of genes undergoing alternative splicing in pathogenic fungi showed their involvement in fungal adaptation to the altered host environment, suggesting a potential role for alternative splicing in fungal pathogenesis. The numbers reported here, as well as the results from recent RNA-sequencing of other fungi could expand and update the data used by [37]. For example, Illumina-based RNA-seq has provided the first evidence of alternative splicing in the pathogen *F. graminearum*, identifying such events at 231 loci [38]. In addition, alternative splicing events were identified at 59% of *Cryptococcus neoformans var. neoformans* loci, many of which were altered in response to changes in the environment [39]. We have previously detected alternative splicing events at 3.6% of *U. maydis* MIPS genes through EST-based analyses [10]. Using the PASA-predicted transcript isoforms, our current dataset, conservatively expands this number in *U. maydis* to ~11%, in *U. hordei* to 9%, and in *S. reilianum* to 7% (Table 2). These findings open avenues for future research in identifying the role of alternative splicing in smut pathogenesis and the regulation of gene expression as a whole.

In addition to updating current MIPS gene models, our sequencing experiments have greatly expanded the annotation depth for each of the smut genomes. Relative to the original annotations in the MIPS database, the number of detected unique transcripts was increased by 2.2 fold for *U. maydis*, 1.5-fold for *U. hordei*, and 1.7 fold for *S. reilianum* (Table 2 and Additional files 7, 8 and 9). The larger increase for *U. maydis* was likely due to sequencing RNA from haploids, dikaryons, and teliospores (dormant and germinating) while only RNA from haploids was sequenced for *U. hordei* and only RNA from haploids and dikaryons for *S. reilianum*. Increased numbers of detected transcripts were also observed by analyses of deep RNA-seq data of *F. graminearum* yielding a 1.2-fold increase and that of *Aspergillus nidulans* yielding a 4.2-fold increase [38, 40]. Among the novel transcripts detected in our study, a small number have putative ORFs. We identified 173, 115, and 45 putative novel protein-coding genes in *U. maydis, U. hordei*, and *S. reilianum*, respectively. Of these putative ORFs, six *U. maydis*, three *U. hordei*, and two *S. reilianum* peptides contain a putative secretion signal as determined by SignalP analyses, suggesting these novel ORFs could correspond to effector proteins (Additional files 10, 11 and 12). In identifying potential effector proteins, it is critical to correctly delineate 5’UTRs. The 5’ UTR data presented here is consistent with earlier 5’ UTR determinations from full-length cDNA analyses [14]; however, it is possible that the data set is still not comprehensive. If this was the case then some starts of transcription may have been missed and this would mean that some effector genes may not have been recognized. As such, the additional effector candidate genes presented here must be considered a minimum number. Even if this was the case, it would not substantially increase the number of predicted proteins or effectors. Other comparable transcriptome studies include updating the genome annotation of *A. nidulans,* in which 29% of the reads mapped to unannotated loci, and of these ~13,000 transcripts, 343 were predicted to code functional proteins based on Pfam/Rfam analyses. This corresponded to a protein-coding gene annotation increase of ~2.9% [40]. Similarly, our expansion of the *U. maydis* protein-coding annotations corresponds to an increase of 2.5%, while *U. hordei* and *S. reilianum* additions correspond to increases of 1.6% and 0.6%, respectively (Additional files 10, 11 and 12). The lower numbers for the latter two smuts are likely due to sequencing fewer libraries relative to *U. maydis.* With more comprehensive RNA-seq for these smuts, the number of updated protein-coding annotations and the number of newly identified transcripts would likely increase further.

### Expansion of the non-coding transcriptome

Although only a small number of the thousands of novel transcripts we detected potentially encode functional proteins, the vast majority do not. These transcripts are, therefore, considered ncRNAs. There is increasing evidence that ncRNAs are functional and there is support for their influence on the expression of other genes. Their functions have been linked to key cellular functions, cancer and even aging [15, 41]. The large numbers of ncRNAs identified here in the smut fungi fall into two categories: lincRNAs and NATs.

#### Long intergenic non-coding RNAs (lincRNAs)

Through RNA-seq analyses, 2414 lincRNAs ≥100 nt were identified in *U. maydis*, 1206 in *U. hordei*, and 1776 in *S. reilianum* (Table 3). All *U. maydis* lincRNAs that were screened for using RT-PCR were detected (13/13; Additional file 20), providing confidence in their existence. These lincRNA numbers are an order of magnitude higher than those identified by RNA-seq studies in other filamentous fungi. Large-scale RNA-seq analyses detected 462 lincRNAs in *Neurospora crassa* [25], 121 lincRNAs in *F. graminearum* [4], 120 lincRNAs in *C. graminicola* [3], and 155 lincRNAs in *R. solani* [21]. Although these studies detected wide-spread transcription of lincRNAs in filamentous fungi, there has been limited functional analysis conducted. In *C. neoformans*, the lincRNA *RZE1* controls yeast-to-hypha morphological transition by regulating a zinc finger transcription factor Znf2 [42] and in *U. maydis*, a lincRNA (*ncRNA1*) influences level of virulence [11], though the mechanism by which *ncRNA1* functions is not yet known. Taken together, these studies indicate that lincRNA transcription is pervasive among the filamentous fungi with the number of lincRNAs substantially higher in the smut fungi. A comprehensive detection of possible lincRNA orthologs among the smut fungi would require complete genome assemblies to enable accurate positional identification of the lincRNAs. This is not currently the case, so our searches were restricted to assessing nucleotide sequence similarity. Among the thousands of *U. maydis*, *U. hordei*, and *S. reilianum* lincRNAs identified here, there was sufficient sequence level conservation to identify 34 potential lincRNA orthologs present in two or all three of the smut fungi. The identification of lincRNA orthologs supports the hypothesis that some of these have functions in the smut fungi.

#### Natural antisense transcripts (NATs)

This report is the first to utilize strand-specific RNA-seq to investigate transcriptomes of smut fungi. The strand-specificity enabled large-scale detection of NATs. Antisense transcription was predicted/detected at 50%/38% of the *U. maydis* loci, 36%/22% of the *U. hordei* loci, and 45%/29% of the *S. reilianum* loci (Table 2). These percentages of protein-coding loci with antisense transcripts are higher than those reported for other filamentous fungi. A recent deep analysis of the *A. nidulans* transcriptome identified at least one antisense read mapped to 72% of annotated loci, however, this was decreased to 14% when only those transcripts with a RPKM values >1 were considered [40] and, in *N. crassa*, antisense transcripts were present at only ~5% of the annotated protein-coding loci [25]. The increased percentage of loci with NATs in *U. maydis* relative to the other smuts likely reflects differences in sequence depth; however, it is noteworthy that *U. maydis* lacks a functional RNAi pathway while *U. hordei* and *S. reilianum* have retained the RNAi machinery [9, 24]. Given that fungal NATs have been shown to undergo cleavage by Dicer (e.g. [25]), we expected that NATs in *U. hordei* and *S. reilianum* would be smaller, on average, due to the presence of functional RNAi machinery. However, the average length of *S. reilianum* NATs (422 bp) was comparable to average *U. maydis* NATs (433 bp), and *U. hordei* NATs were longer on average (538 bp) (Additional files 17, 18 and 19). These findings suggested that if the detected NATs are functional in these species, they likely exert their functions without involvement of an RNAi pathway.

The functional characterization of NATs in filamentous fungi has identified roles in development, metabolism, and pathogenesis (Reviewed in [18]). In the mushroom-forming basidiomycete *Coprinopsis cinerea*, a large number of antisense transcripts were identified, and a substantial subset of these NATs have expression patterns consistent with their having a role in fruiting body development, although clear functional analysis remains to be carried out [16]. Roles for NATs in regulating cellular metabolism stem from studies in *N. crassa* and *U. maydis*. In *N. crassa*, light-sensitive NATs regulate the circadian rhythm [43, 44]. The core oscillator gene frequency (*frq*) and a long-non-coding antisense to *frq*, *qrf*, regulate each other’s expression through transcriptional interference in a negative feedback loop that involves chromatin modifications and premature transcriptional termination [43]. In *U. maydis*, a teliospore-specific NAT (*as-ssm1*) to the mitochondrial seryl-tRNA synthetase (*ssm1*) regulates the stability of *ssm1* mRNA through its direct binding, and also influences growth rate, mitochondrial membrane potential, and oxygen consumption rates [19]. These data led to the presentation of a model in which *as-ssm1* expression in the dormant teliospore (1) contributes to the suppression of mitochondrial function during dormancy through direct binding of *ssm1* and (2) preserves *ssm1* mRNAs for rapid translation upon germination. An indication of a role for NATs in regulating pathogenesis comes from investigating the bidirectional regulatory region that controls the expression of teliospore-specific NATs to a *U. maydis* xylitol dehydrogenase (*UMAG_02150*) and a D-gluconic acid reductase (*UMAG_02151*) gene, as well as *ncRNA1* [11, 15]. Deletion of this regulatory region altered the expression of the NATs and *ncRNA1* and significantly reduced pathogenesis. This indicated that proper control of NAT expression is required for full pathogenesis. The details of how the altered regulation impacts pathogenesis are under investigation (Goulet et al., unpublished). It is likely that there are other functional antisense present in the teliospore. RNA-seq detected 116 teliospore-specific NATs, 164 haploid cell-specific NATs, and 164 dikaryon-specific NATs. Confirming cell-type-specific expression of these NATs will identify candidates for future functional investigation.

Given the widespread existence of NATs in filamentous fungi and their emerging functional relevance [18, 45, 46], it is anticipated that a large number of the NATs we have detected in this study are functional. Interestingly, ~50% of the *U. maydis* NATs are conserved in either *U. hordei* (~15%), *S. reilianum* (~22%) or both (~13%) (Fig. 1). In vitro analyses confirmed the presence of 18/18 *U. maydis* NATs that were screened, several of which were predicted to contain introns, overlap known effector proteins or be expressed in a cell-type-specific manner. Conservation between species and expression in specific cell-types are characteristics which point to functional relevance [47]. The identification of such a high proportion (nearly one fifth) of genes with conserved NATs suggests their potential involvement in something of broad influence within the smut fungi. As an initial functional characterization, we conducted GO term enrichment analyses on the NATs conserved among the smut fungi. The analyses revealed that the NATs conserved among all three smut fungi are complementary to genes that are enriched in the molecular function categories: binding, DNA binding, nucleoside triphosphatase activity, and its sub-category helicase activity. This indicates that genes in these categories in the smut fungi have homologous complementary antisense transcripts. If the presence of these NATs is independently confirmed in all species, it could indicate a common mechanism of modulating the expression of these genes. Since the genes in the DNA binding and helicase activity categories alone include histones, transcription factors, chromatin modification enzymes, proteins involved in DNA replication including polymerase subunits, as well as DNA and RNA helicases, this enriched group of genes have the potential to influence gene expression at multiple levels. Therefore, if the expression of these genes is modulated by antisense, then antisense could influence multiple levels of gene expression control and through this, practically all aspects of fungal development. Further, as stated earlier, these conserved NATs must be functioning in an RNAi-independent manner. Interestingly, the GO analysis also provides a basis for a hypothesis regarding how this might occur. Our initial hypotheses are that 1) NATs influence the expression of genes that can modulate gene expression and 2) this NAT influence is modulated through a feedback mechanism involving NAT control of protein expression and protein control of NAT expression. Components of these hypotheses are currently being investigated using NATs and helicases preferentially expressed in the teliospore. As such, this RNA-seq analysis has substantially advanced the knowledge of a model organism and provided the basis for investigating NAT-mediated regulation as a new level of gene expression control in phytopathogenic fungi.

## Conclusions

In the present study, we have expanded and updated the genome annotations for three closely related smut fungi, *U. maydis*, *U. hordei*, and *S. reilianum*. We have updated incomplete or incorrect MIPS gene models, and have added information regarding UTR lengths and alternative splicing. Hundreds of novel putative protein-coding genes have been identified across the three genomes, a handful of which may represent effector proteins. Finally, our analyses enabled the detection of thousands of lincRNAs and NATs in these smuts, and the determination that a high proportion of the NATs are conserved among the three fungi. This provides the basis of hypotheses regarding NAT function and a starting point for functional analyses, which may reveal important roles for ncRNAs in these species.

## Methods

### Fungal strains, growth conditions and cultivation of cell-types

All fungal strains used in this study are listed in Table 1. *U. maydis* and *S. reilianum* cultures were incubated at 28 °C and *U. hordei* cultures were incubated at 22 °C, unless otherwise noted. Haploid *U. maydis* strains 518 and 521, *U. hordei* strains Uh364 and Uh365, and *S. reilianum* strains SRZ1 and SRZ2 were inoculated from frozen stocks onto potato dextrose agar (PDA) plates. Single colonies from each plate were incubated in 3 mL potato dextrose broth (PDB) shaking at 250 rpm. After ~48 h, cellular concentrations were normalized to an OD_600_ of 1.0, and 10 μL of each haploid strain was spotted on PDA plates supplemented with 2% activated charcoal (10 spots per plate, 8 plates per strain), and incubated at room temperature (RT). 518 × 521 and SRZ1 × SRZ2 forced dikaryon growth was induced under similar conditions, as described in [11]. Briefly, haploid cultures were grown overnight as described above and diluted with sterile water to an OD_600_ of 1.5. Equal volumes of compatible haploid cultures were combined in a 1.5 mL tube so that 10 μL of each mixture could be spotted on PDA plates supplemented with 2% activated charcoal (10 spots per plate, 8 plates per strain). After 2 days of growth, haploid and dikaryon colonies were inspected visually for the presence or absence of filamentous growth. Colony morphology was confirmed and documented using a Leica S8 APO stereo microscope (Leica Microsystems), as described in [48]. Haploid or dikaryon cells were scraped from the medium with a scoopula, immediately frozen in liquid nitrogen, and stored at −80 °C until RNA isolation. *U. maydis* 518 × 521 teliospores were harvested from mature tumours of corn (*Z. mays* L. ‘Golden Bantam’) as described in [49] with the following changes: overnight haploid cell cultures were diluted with sterile water to an OD_600_ of 1.0, equal volumes of compatible haploids were combined in 50 mL conical tubes, and 6 mL of this mixture was injected down the cob silk shaft. Harvested teliospores were germinated in PDB + Streptomycin (160 μg/mL) shaking 90 rpm. Teliospores were sampled at 0 h, 9 h, and 18 h post induction of germination (PIG). Teliospore germination was observed at 400X magnification using a Axio Scope.A1 compound microscope (Carl Zeiss MicroImaging), as described in [48]. For RT-PCR experiments, in addition to the cell-types described above, 7-day-old seedlings were infected with 518 × 521, as described in [1]. *U. maydis*-infected plant material was harvested at 8 and 14 days post infection (DPI), immediately frozen in liquid nitrogen, and stored at −80 °C until RNA isolation.

### RNA extraction, cDNA library preparation and RNA-sequencing

Total RNA was isolated, precipitated, and DNase I-treated following methods outlined in [11]. Briefly, harvested cells were ground in liquid nitrogen using a mortar and pestle. The ground cells or teliospores were resuspended in TRIzol reagent (Life Technologies) and transferred to 2 mL screw-cap tubes containing Lysing Matrix C (MP Biomedicals). Cells were disrupted as described by [49] using a bead-mill and RNA was isolated following the manufacturer’s protocol. RNA was treated with DNAseI (New England Biolabs) to remove contaminating DNA. RNA was precipitated using RNA precipitation solution and isopropanol [50], washed with 75% ethanol, and resuspended in nuclease-free water (Life Technologies). RNA quality was confirmed through electrophoresis of glyoxalated RNA on a denaturing agarose gel (1X BPTE) as outlined in [50]. Total RNA was sent to the Clinical Genomics Centre at Mount Sinai Hospital (Toronto, Canada). RNA quality was assessed using a Bioanalyzer (Agilent Technologies) and all samples had an RNA integrity number (RIN) greater than 7.5. Poly(A) mRNA was enriched using oligo dT-beads and cDNA libraries were prepared using the TruSeq Stranded mRNA Library Preparation kit (Illumina Inc.). Barcoded libraries were pooled in equimolar quantities and two libraries were sequenced per lane on a HiSeq 2500 System (Illumina Inc.) to generate 75 bp paired-end reads.

### RNA-sequencing read alignment, transcript characterization, and gene ontology enrichment

For each fungal species, the resulting sequences were pooled and then assembled into *de novo* transfrags using Trinity [26] with the following settings: −-SS_lib_type RF, −-min_kmer_cov = 2, −-min_contig_length = 100, −-normalize_reads, and --jaccard_clip. Genome-guided Trinity transfrags were also predicted with the following additional setting: −-genome_guided_max_intron = 1500. The published genome annotations [1, 8, 9] were used as references by the program to assemble spliced alignments (PASA; [27]), which created updated gene models for each fungus using the *de novo* and genome-guided transfrag assemblies. These predicted gene models were then categorized with Cufflinks Cuffcompare [28]. For each library, paired-end reads were aligned to their respective genome sequence using CLC Genomics Workbench v9.0 (Qiagen) with the following changes to default settings: mapping type = map to gene regions only, strand specific = reverse, count paired reads as two = no, expression value = FPKM. This resulted in the calculation of fragments per kilobase of transcript per million mapped reads (FPKM) values by CLC Genomics Workbench for each transcript. Transcripts were considered to be specifically expressed in a cell-type if they had an FPKM > =1 in one cell-type and <1 in others. For haploid cells FPKM > =1 were required in both the 518 and 521 libraries. For dormant teliospores, FPKM > =1 was required for the T00 library (and not the T09 or T18 germinating teliospore libraries). We used portions of the Trinotate annotation suite (https://trinotate.github.io/) to further characterize novel transfrags, including: BLASTp sequence similarity search to known sequence data (SwissProt database, August 2014), RNAmmer v1.2 [51], and protein signal peptide prediction (SignalP v4.0) [52]. Similarity Matrix of Proteins (SIMAP) values have previously been used to identify paralogs between smut species [9], and using a smaller dataset, [53] identified putative fungal orthologs using a reciprocal best-BLASTp hit approach. In this study, the two methods were combined and orthologs were identified as reciprocal best SIMAP hits. When calculating the number of orthologous antisense in Fig. 1, the merged transcripts were excluded because they could be Trinity-related artifacts resulting from the compact fungal genomes that may contain overlapping UTRs. For *U. maydis* genes, gene ontology term enrichment [54] was assessed using the web-based g:Profiler [55] with a g: SCS significance threshold <0.05. To identify lincRNAs with similar sequences among the smut fungi, in-house BLAST databases were created from the updated gene models described above; therefore, these databases contained both coding and non-coding nucleotide sequences. BLAST 2.5.0+ [56] was performed with an e-value cutoff of 1E-03 to identify class ‘p’ and class ‘u’ transcripts with similar sequence composition in the other two smuts.

### Transcript expression analysis

Genome sequences from the MIPS *U. maydis* database (MUMDB; [57]) were used to design strand-specific first-strand synthesis primers, as well as PCR primers, as described in [15, 58]. All primers used in this study are listed in Additional file 21. Antisense strand-specific first-strand synthesis primers were designed based on RNA-seq-predicted transcript structures. First-strand synthesis primers included oligo(dT)_16_, DEPC-treated H_2_O, a sense-specific first-strand primer or an antisense-specific first-strand primer. First strand synthesis was conducted on 200 ng of DNase I-treated RNA, using the TaqMan Gold RT-PCR kit (Applied Biosystems) following methods outlined in [58]. cDNA was diluted eightfold (1:7) with dH_2_O. PCRs were conducted using the AmpliTaq Gold DNA Polymerase Kit (Applied Biosystems) following the manufacturer’s recommended protocol. One third of the PCR products were visualized using agarose gel electrophoresis (1X TAE) and were subsequently stained with 0.3 μg/mL ethidium bromide (BioShop).

## Additional files

Additional file 1: Microscopic observation of fungal cell morphology. (TIF 2503 kb)

Additional file 2: RNA-seq library information. (XLSX 316 kb)

Additional file 3: *U. maydis* GFF3-formatted annotation file. (TXT 12298 kb)

Additional file 4: *U. hordei* GFF3-formatted annotation file. (TXT 11903 kb)

Additional file 5: *S. reilianum* GFF3-formatted annotation file. (TXT 10146 kb)

Additional file 6: RNA-seq based detected gene models using different FPKM cut-off values. (XLSX 239 kb)

Additional file 7: *U. maydis* RNA-seq based predicted gene models. (XLSX 661 kb)

Additional file 8: *U. hordei* RNA-seq based predicted gene models. (XLSX 644 kb)

Additional file 9: *S. reilianum* RNA-seq based predicted gene models. (XLSX 644 kb)

Additional file 10: Trinotate results for *U. maydis* intergenic transcripts. (XLSX 251 kb)

Additional file 11: Trinotate results for *U. hordei* intergenic transcripts. (XLSX 247 kb)

Additional file 12: Trinotate results for *S. reilianum* intergenic transcripts. (XLSX 242 kb)

Additional file 13: BLASTn results find intergenic transcripts with similar sequences in smuts. (XLSX 285 kb)

Additional file 14: *U. maydis* predicted antisense and putative orthologs with predicted antisense. (XLSX 436 kb)

Additional file 15: *U. hordei* predicted antisense and putative orthologs with predicted antisense. (XLSX 392 kb)

Additional file 16: *S. reilianum* predicted antisense and putative orthologs with predicted antisense. (XLSX 417 kb)

Additional file 17: *U. maydis* detected antisense and putative orthologs with detected antisense. (XLSX 386 kb)

Additional file 18: *U. hordei* detected antisense and putative orthologs with detected antisense. (XLSX 332 kb)

Additional file 19: *S. reilianum* detected antisense and putative orthologs with detected antisense. (XLSX 351 kb)

Additional file 20: RT-PCR results supporting *in silico* analysis. (XLSX 242 kb)

Additional file 21: Primers used in this study. (XLSX 244 kb)

## Abbreviations

DPI: Days post infection
EST: Expressed-sequence tag
FPKM: Fragments per kilobase of transcript per million mapped reads
lincRNA: Long intergenic non-coding RNA
NAT: Natural antisense transcript
ncRNA: Non-coding RNA
PIG: Post induction of germination
RIN: RNA integrity number
RPKM: RPKM Reads per kilobase of transcript per million mapped reads
RT: Room temperature
UTR: Untranslated region
