# Supplementary figures and images for: Transcriptome analysis of smut fungi reveals widespread intergenic transcription and conserved antisense transcript expression
Source: BMC Genomics. 2017 May 2;18:340. doi: 10.1186/s12864-017-3720-8 (PMC5414199; doi:10.1186/s12864-017-3720-8)

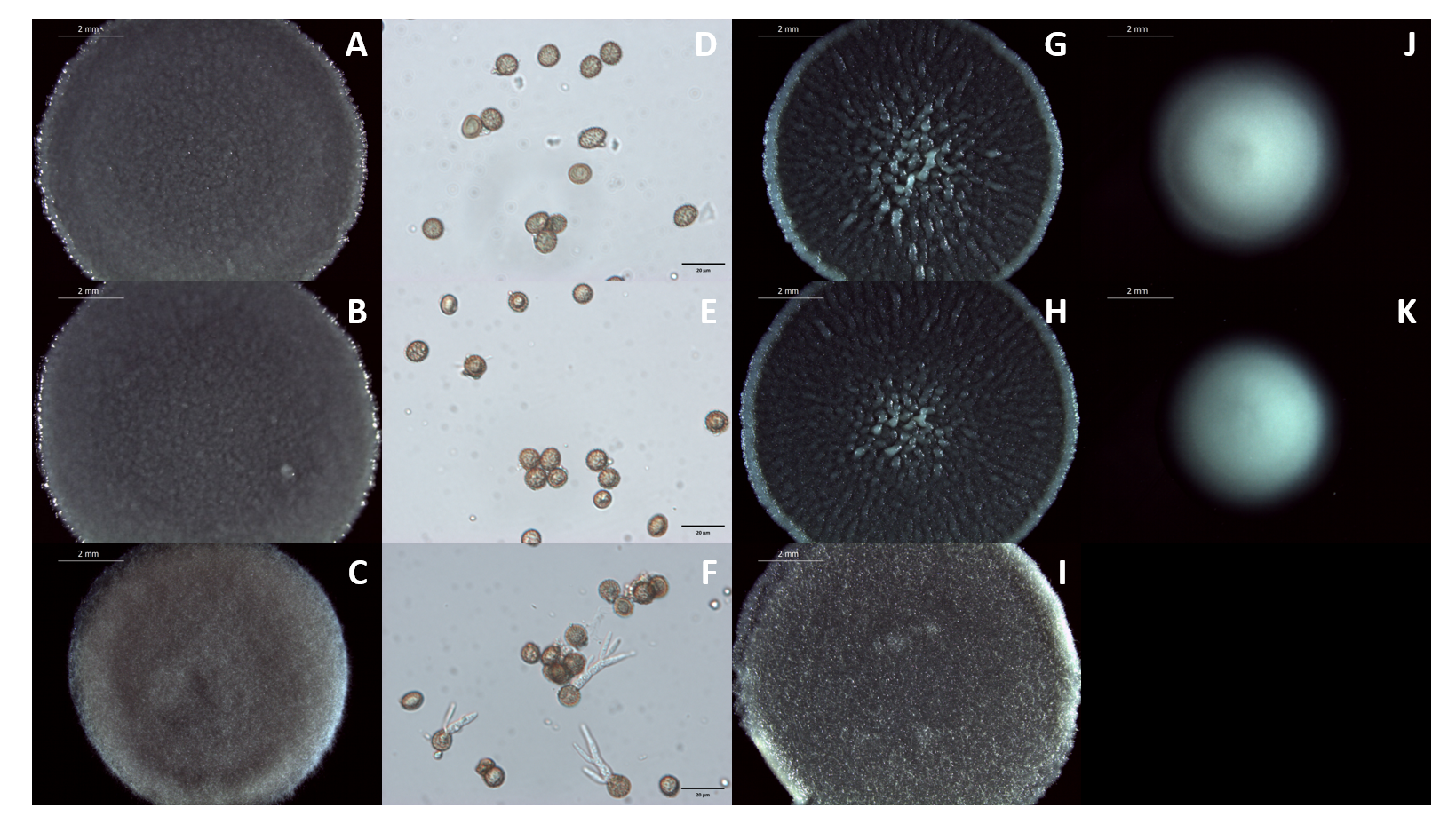

Supplement: Supplementary file 1 — Microscopic observation of fungal cell morphology. (TIF 2503 kb) [file 12864_2017_3720_MOESM1_ESM.tif]
